# Comparing a Portable Motion Analysis System against the Gold Standard for Potential Anterior Cruciate Ligament Injury Prevention and Screening

**DOI:** 10.3390/s24061970

**Published:** 2024-03-20

**Authors:** Nicolaos Karatzas, Patrik Abdelnour, Jason Philip Aaron Hiro Corban, Kevin Y. Zhao, Louis-Nicolas Veilleux, Stephane G. Bergeron, Thomas Fevens, Hassan Rivaz, Athanasios Babouras, Paul A. Martineau

**Affiliations:** 1 Faculty of Medicine and Health Sciences, McGill University, 3605 Rue de la Montagne, Montreal, QC H3G 2M1, Canada; nicolaos.karatzas@mail.mcgill.ca (N.K.); patrik.abdelnour@mail.mcgill.ca (P.A.); kevin.zhao2@mail.mcgill.ca (K.Y.Z.); 2 Division of Orthopaedic Surgery, McGill University Health Centre, 1650 Cedar Ave, Montreal, QC H3G 1A4, Canada; jason.corban@mail.mcgill.ca; 3 Shriners Hospital for Children—Canada Motion Analysis Centre, 1003 Decarie Blvd, Montreal, QC H4A 3J1, Canada; lnveilleux@shrinenet.org; 4 Department of Surgery, McGill University, 1001 Décarie Blvd, Montreal, QC H4A 3J1, Canada; 5 Department of Orthopaedic Surgery, Jewish General Hospital, 3755 Chem. de la Côte-Sainte-Catherine, Montreal, QC H3T 1E2, Canada; 6 Department of Computer Science and Software Engineering, Concordia University, 1455 De Maisonneuve Blvd. W., Montreal, QC H3G 1M8, Canada; thomas.fevens@concordia.ca; 7 Department of Electrical and Computer Engineering, Concordia University, 1455 De Maisonneuve Blvd. W., Montreal, QC H3G 1M8, Canada; hassan.rivaz@concordia.ca; 8 Department of Experimental Surgery, McGill University, 845 Sherbrooke St W, Montreal, QC H3A 0G4, Canada; athanasios.babouras@mail.mcgill.ca; 9 Department of Health, Kinesiology and Applied Physiology, Concordia University, 1455 Blvd. De Maisonneuve Ouest, Montreal, QC H3G 1M8, Canada

**Keywords:** kinematics, injury prevention, ACL, biomechanics, motion analysis

## Abstract

Knee kinematics during a drop vertical jump, measured by the Kinect V2 (Microsoft, Redmond, WA, USA), have been shown to be associated with an increased risk of non-contact anterior cruciate ligament injury. The accuracy and reliability of the Microsoft Kinect V2 has yet to be assessed specifically for tracking the coronal and sagittal knee angles of the drop vertical jump. Eleven participants performed three drop vertical jumps that were recorded using both the Kinect V2 and a gold standard motion analysis system (Vicon, Los Angeles, CA, USA). The initial coronal, peak coronal, and peak sagittal angles of the left and right knees were measured by both systems simultaneously. Analysis of the data obtained by the Kinect V2 was performed by our software. The differences in the mean knee angles measured by the Kinect V2 and the Vicon system were non-significant for all parameters except for the peak sagittal angle of the right leg with a difference of 7.74 degrees and a *p*-value of 0.008. There was excellent agreement between the Kinect V2 and the Vicon system, with intraclass correlation coefficients consistently over 0.75 for all knee angles measured. Visual analysis revealed a moderate frame-to-frame variability for coronal angles measured by the Kinect V2. The Kinect V2 can be used to capture knee coronal and sagittal angles with sufficient accuracy during a drop vertical jump, suggesting that a Kinect-based portable motion analysis system is suitable to screen individuals for the risk of non-contact anterior cruciate ligament injury.

## 1. Introduction

Preventative measures including neuromuscular training and injury prevention programs, such as the FIFA11+, target biomechanical patterns to decrease the overall risk of anterior cruciate ligament (ACL) injury [1,2]. Given the success of such programs, the identification of individuals who are at risk of ACL injury is of great significance to athletes and to the healthcare sector as a whole.

ACL injuries that occur without physical contact with another athlete or object are considered non-contact ACL injuries. Seventy percent of all ACL injuries occur by this mechanism, making it an important target for overall ACL injury prevention [3,4]. Individuals with poor neuromuscular control of their lower limb biomechanics are at a particularly increased risk of these injuries due to their decreased ability to dynamically stabilize their knee during periods of high stress, such as during pivoting and landing [5,6]. This was highlighted in a study conducted by Hewett et al. [5] that prospectively assessed the knee kinematics of 205 female varsity athletes in a motion analysis laboratory. This investigation demonstrated a correlation between non-contact ACL injury and greater initial coronal (IC), greater peak coronal (PC), and smaller peak sagittal (PS) knee angles during a standardized drop vertical jump (DVJ) [5]. Although this investigation highlighted important biomechanical risk factors and potential targets for sport-specific neuromuscular training, traditional motion analysis labs have significant barriers to their routine use in the clinical setting, including a lack of portability and extensive testing time by trained personnel [6].

More recently, our group published a separate study testing a portable and cost-effective Kinect V2 motion capture system (Microsoft, Redmond, WA, USA) as a screening modality for ACL injury risk in 102 university varsity athletes [7]. We found that the IC, PC, and PS angles during a DVJ all demonstrated a good to excellent prognostic value for non-contact ACL injury through receiver operating characteristic analysis. Additionally, an investigation by Gray et al. [8] demonstrated that the Kinect-based portable system could be used to measure knee ankle separation ratios (KASRs), as a proxy for knee valgus, with a similar degree of accuracy to the Vicon motion capture system. These findings substantiate the need for the validation of low-cost practical motion capture devices like the one used in our study for assessing DVJ parameters in the context of non-contact ACL injury [7].

The purpose of the present study was to compare the Kinect V2 to the current gold standard Vicon system (Vicon, Los Angeles, CA, USA) for assessing drop vertical jump parameters. Specifically, we assessed the initial coronal (IC), peak coronal (PC), and peak sagittal (PS) angles of the knee. It was hypothesized that the Kinect V2 will be able to measure these angles with a similar degree of accuracy to that of the Vicon system.

## 2. Materials and Methods

### 2.1. Study Participants

This investigation was a descriptive laboratory study. Ethics approval was received from the Research Ethics Office of the Faculty of Medicine and Health Sciences of McGill University (Montreal, QC, Canada) prior to the start of this investigation and informed consent was obtained from all participants in this study. Exclusion criteria included individuals with a lower limb injury at the time of consent, individuals aged less than 18 years old, and individuals aged more than 30 years old. The demographic characteristics of the participants are summarized in Table 1.

### 2.2. Motion Analysis

**Figure 1:**
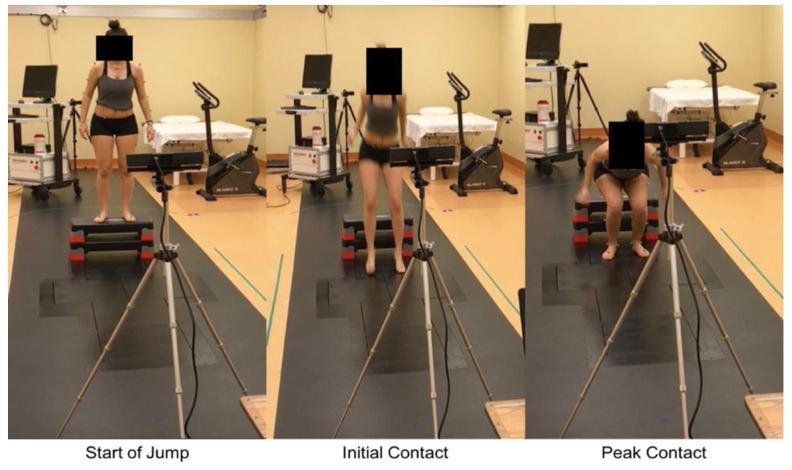
Images of the Kinect V2 setup with freeze frames from the beginning of the DVJ as well as the two significant time points: initial and peak contact. The Kinect V2 was mounted on a tripod at a distance of 2.5 m from the participant for optimal accuracy.

**Figure 2:**
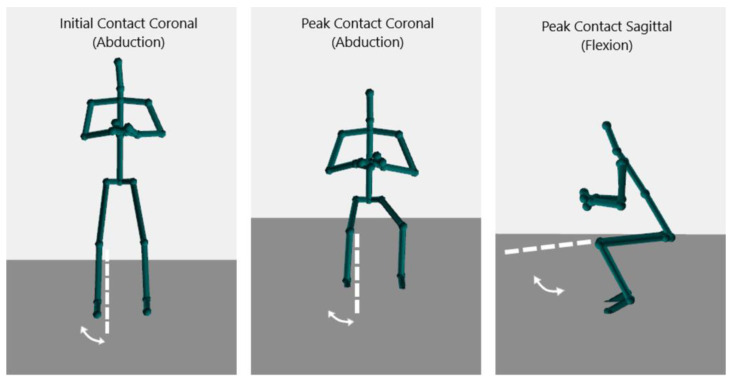
Images of the of the output from the Kinect V2 demonstrating the initial coronal, peak coronal, and peak sagittal angles during a DVJ.

Knee kinematics were assessed during a standardized DVJ off a 31 cm box (Figure 1). Each participant performed three DVJs and the IC, PC, and PS angles were captured by both the Kinect V2 and Vicon systems simultaneously (Figure 2). Participants were instructed to stand with their toes off the edge of the box, lean forward with their chest such that they fall off the box, land on both feet simultaneously, and to jump as high as possible upon making first contact with the floor.

A Plug in Gait model (PiG) was used with the Vicon system. This required 34 markers to be placed on each participant and employs 10 cameras capable of capturing at a frame rate of 120 Hz. For each participant’s captured DVJs, their skeletal model was labeled and reconstructed in order to extract quantifiable knee angles using Mokka (Motion Kinematic and Kinetic Analyzer, Biomechanical ToolKit, QC, Canada, version 0.6.2) [9]. IC, PC, and PS angles were extracted for analysis.

The Kinect V2, programmed using an open-source software development kit (Microsoft, Redmond, WA, USA, SDK version 2.0), was mounted on a tripod 2.5 m away from the athletes (Figure 1). The system is capable of three-dimensional tracking with an infrared depth sensor at a capture rate of 30 Hz [8]. Our software used specific formulas to calculate the angles of interest; force vectors from the knee to the hip were used to represent the femur, and from the knee to the ankle to represent the tibia. These formulas were used to calculate coronal and sagittal knee angles at every frame of the DVJ (Equation (1)). The coronal knee abduction and sagittal knee flexion angles were then calculated (Equations (2) and (3)). The pertinent frames from which the IC, PC, and PS angles were extracted were determined manually by an author blinded to injury outcomes (NK) based on kinematic triggers, which are specific defined movements. The kinematic trigger used to detect the initial landing frame was when the foot joint stopped travelling in a downward direction, indicating that the subject made contact with the floor. The kinematic trigger used to detect the peak landing frame was the moment at which the ankle joint and hip joint were closest to each other. A data file with the IC, PC, and PS angles was then generated using Microsoft Excel (Microsoft, Redmond, WA, USA, version 16.0) for statistical analysis.

Equation (1): Vector definition of the femur and tibia:

(1) $${\vec{tibia}=P}_{knee}-P_{hip}\;{\vec{femur}=P}_{knee}-P_{ankle}$$

Equation (2): Formula for calculating coronal knee abduction angles:

(2) $$\begin{array}{c} x=RotationAxis.x\times \sin\left(RotationAngle/2\right)\\ y=RotationAxis.y\times \sin\left(RotationAngle/2\right)\\ z=RotationAxis.z\times \sin\left(RotationAngle/2\right)\\ w=\cos\left(RotationAngle/2\right) \end{array}$$

Equation (3): Formula for calculating sagittal knee flexion angles:

(3) $$\theta_{sagittal}=180{}^{\circ}-\mathrm{acos}\left(\frac{\vec{femur}\times \vec{tibia}}{∥\vec{femur}∥∥\vec{tibia}∥}\right)$$

### 2.3. Statistical Analysis

The Shapiro–Wilk test was used to test for the normality of each parameter. For normally distributed parameters, the independent-samples *t*-test was used to test for statistically significant differences in the mean angles measured by the Kinect V2 and the Vicon system. Non-normally distributed parameters were compared using the Wilcoxon signed-rank test. The *t*-test was chosen because it utilizes the full information of our data, including means and variances, and offers a potentially higher power compared to the non-parametric tests when we have normal distribution. The Wilcoxon signed-rank test was best suited for our analysis given the small sample size, as it is less sensitive to potential outliers that might affect the power in tests based on raw data means. Additionally, the rank test helps avoid issues with power and Type I error inflation, which might occur with non-normal data. In both cases, we acknowledge that the small sample size is a limitation that can affect the power to detect true effects.

The intraclass correlation coefficient (ICC; two-way mixed-effects model, single measures, absolute agreement) was used to assess the level of agreement between the Kinect V2 and the Vicon system for the IC, PC, and PS angles. ICC values greater than 0.75 have been shown to indicate excellent inter-rater reliability in the context of kinematic assessment [8]. Statistical significance was set at *p* < 0.05. All statistical analyses were performed using statistical software (SPSS Version 27; IBM, Armonk, NY, USA).

## 3. Results

Mean IC, PC, and PS angles were identified for the left and right knees separately for each participant, amounting to six parameters for analysis. Each of the 11 participants completed three DVJs, each measured by both Kinect V2 and Vicon simultaneously, amounting to 66 data points per parameter.

### 3.1. Comparison of Means

Three datasets had non-normal distributions: the left PC angles measured by the Kinect V2 (W = 0.86, *p* < 0.01) and the Vicon system (W = 0.89, *p* < 0.01), and the right PS angles measured by the Vicon system (W = 0.93, *p* = 0.036). All other parameters were normally distributed. The mean angles measured by the Kinect V2 and the Vicon system, along with the mean differences, are presented in Table 2. The mean IC and PC angles measured by the two systems differed by a maximum of 0.62 degrees. The mean PS angles differed with the Kinect, which identified increased angles of 8.35 and 7.74 degrees for the left and right knees, respectively. Out of the six parameters, only the mean right PS angle was significantly different between the Kinect V2 and the Vicon system with an observed difference of 7.74 degrees and a *p*-value of 0.008.

### 3.2. Assessment of Agreement

An ICC analysis for the assessment of the level of agreement between the Kinect V2 and the Vicon system demonstrated coefficient values that were consistently over 0.75 for all six parameters, as outlined in Table 3.

### 3.3. Visual Assessment

**Figure 3:**
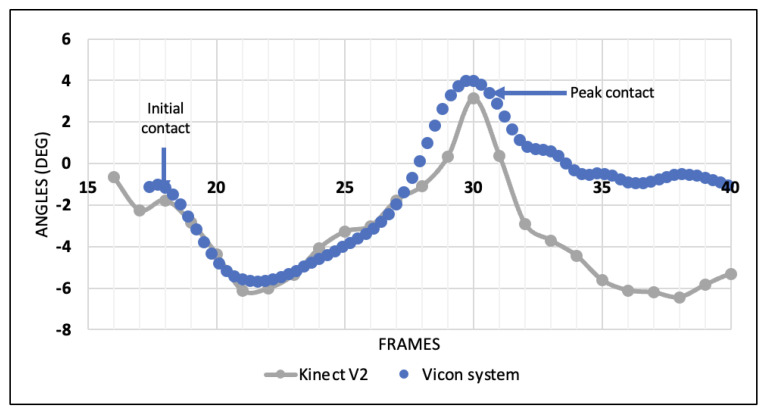
Coronal knee angles per frame of a healthy individual during a DVJ measured by the Kinect V2 and the Vicon system.

**Figure 4:**
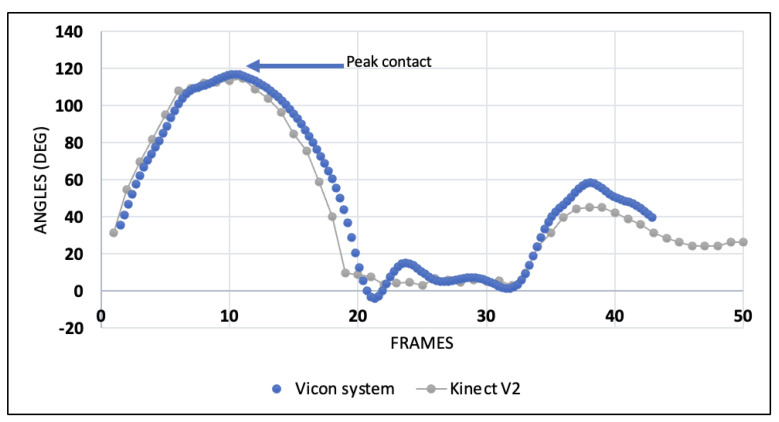
Sagittal knee angles per frame of a healthy individual during a DVJ measured by the Kinect V2 and the Vicon system.

Graphical representations of the frame-by-frame angles measured for a single DVJ by both the Kinect V2 and the Vicon system are presented in Figure 3 and Figure 4 for the coronal and sagittal angles, respectively. In the coronal plane, there is a moderate degree of angle variation between frames and between the two systems; however, the concordance at the frames of interest (initial contact and peak contact) is high. Figure 4 reveals a high degree of concordance between the two systems for tracking sagittal angles throughout all frames of the DVJ.

## 4. Discussion

There were no statistically significant differences in the IC and PC angles, as well as the PS angles of the left knee, that were measured by the Kinect V2 and the Vicon system. The right knee PS angles measured by the two systems were statistically different. The ICC analysis revealed excellent agreement between the two systems for assessing the IC, PC, and PS angles of both knees.

### 4.1. Existing Literature and Related Kinematic Assessments

This is the first investigation to directly assess the accuracy of the Kinect V2 for measuring the IC, PC, and PS angles of the DVJ. Nonetheless, there has been a growing body of literature on the accuracy of Kinect systems for various other kinematic assessment applications [8,10,11,12,13]. One such investigation evaluated the accuracy of the Kinect V2 compared to the Vicon system for assessing the knee–ankle separation ratio (KASR) at the initial contact and peak flexion of a DVJ. The KASR is a proposed method of assessing the degree of knee valgus throughout a DVJ by using the distance between the knee and ankle in the coronal plane. In their study, the Kinect V2 demonstrated excellent correlation compared to the Vicon system (ICC > 0.75) for these coronal plane measures. While their study offered valuable insight into the motion-tracking potential of the Kinect V2, it did not directly assess coronal knee angles at the time points of interest in this study, and assessment of knee kinematics in the sagittal plane was not performed. This study provided a more granular and focused assessment of the Kinect V2, including quantifying its accuracy in tracking sagittal knee angles. Our research further contributes by examining and analyzing the sagittal planes of the jump, which was not previously studied.

Another study by Hewett et al. of 205 female athletes, as discussed previously, highlighted the association between increased IC and PC angles and decreased PS angles during a DVJ with a greater risk of non-contact ACL injury [5]. Their study used an optoelectronic system, similar to the Vicon system used in this study. These marker-based motion capture systems employ costly, high-speed motion analysis cameras that can cost up to $150,000 and take up to 4 h of analysis to obtain meaningful results [8,14]. These challenges impeded the transfer of such findings into real-life clinical applications; as such, there existed a need to identify alternative motion capture systems that were low-cost, robust, clinician-friendly, and able to track pertinent DVJ parameters with reliable accuracy. Our presented data bring to light low-cost alternatives that could be ground-breaking in providing reliable and affordable biomechanics analysis.

More recently, our group used the Kinect V2, a motion capture system that meets such criteria, to assess the same DVJ parameters as risk factors for non-contact ACL injury in 102 male and female university varsity athletes [7]. In this study, increased PC and decreased PS angles were significantly associated with non-contact ACL injury, and ROC analysis demonstrated a good to excellent prognostic ability of the IC, PC, and PS angles for ACL injury risk assessment [7]. The findings of this investigation suggest that the Kinect V2 could fill the growing niche of kinematic-based injury risk assessment. As such, there emerged a need for an objective lab-based validation of this system, specifically for assessing DVJ parameters, to support the continued development of this potential modality for widespread ACL injury risk screening.

### 4.2. Accuracy of Kinect V2 Compared to Vicon System

We first compared the means of each DVJ parameter as measured by the Kinect V2 and the Vicon system, with the hypothesis that there would not be any statistically significant differences. There was no significant difference in the IC and PC angles of both knees as measured by the two systems, suggesting that the Kinect V2 measures the coronal knee angles of the DVJ in a similar manner compared to the gold standard Vicon system. The left IC angles were also not significantly different between the two systems. Out of the six parameters, only one (the right PS angle) was statistically different with a mean difference of 7.74° between the two systems. Interestingly, the left PS angle differed by 8.35° between the two systems; however, this difference was not statistically significant. We hypothesize that this may be due to a difference in how the data were spread for the two parameters. While the statistical tests employed for these analyses (the independent-samples *t*-test and Wilcoxon signed-rank test) do not actually classify the level of agreement between the Kinect V2 and the Vicon system, these findings may suggest that the Kinect V2 is more accurate at assessing coronal knee angles in comparison to sagittal knee angles. In our study, it is possible that the Kinect V2 was less reliable for assessing the peak sagittal angle than for assessing coronal angles because the DVJ consists of substantially faster movement compared to the speed of movement in gait analysis, and because there is a greater range of variability in sagittal knee angles compared to coronal knee angles during a DVJ. Whether the decreased accuracy in the tracking of sagittal knee angles is of clinical significance is unclear. Of note, in our Kinect-based investigation of DVJ parameters as ACL injury risk factors, the mean PS angle was 17.84° smaller in ACL-injured athletes compared to non-injured athletes. This is over double the mean differences in PS angles measured between the Kinect V2 and the Vicon system in this study, suggesting that these differences may not bear significant clinical value. Nonetheless, since the mean PS angles measured by the Kinect V2 were smaller than those of measured by the Vicon system, this may be a potential target for improvement for the Kinect V2 in future updates or iterations.

Intraclass correlation coefficient analysis was used to provide a more in-depth assessment of the actual level of agreement between the two systems on each DVJ parameter. For the 33 jumps assessed in this investigation, the ICC values for the IC, PC, and PS angles of the left and right knee ranged from 0.771 to 0.917. These ICC values are all well above the established cut-off for excellent inter-observer reliability for kinematic assessment that has been reported in the literature [8]. Interestingly, while all ICC values were within the range of excellent agreement, the ICC values for left and right PS angles were lower than the rest (0.771 and 0.770, respectively). In conjunction with the findings discussed above, these results may suggest that there is less objective agreement between the two systems for assessing PS angles of the DVJ; however, these differences are not necessarily functionally or clinically significant. Overall, these findings highlight the potential for the Kinect V2 to provide accurate biomechanical assessment during a DVJ, particularly as a screening tool in the context of ACL injury risk assessment.

### 4.3. Kinect V2 for ACL Injury Risk Assessment

The ease of use of the Kinect V2, coupled with its acceptably high degree of accuracy, has resulted in its increased use in the rehabilitation sphere [15]. A review conducted by Mousavi and Khademi found that while the Microsoft Kinect does not have absolute precision, it is acceptably accurate for rehabilitation purposes [15]. These findings are in line with the results of our current investigation. The margin of error associated with the Kinect V2 is highlighted by the variability seen in its measurements in the coronal plane (Figure 3). In contrast to the measurement pattern of the Vicon system, the Kinect V2’s measurement pattern displays a higher degree of frame-to-frame variability throughout the course of the DVJ when looking at coronal knee angles. This decreased precision is consistent with the findings of previous investigations [10,16]. Interestingly, in contrast to findings of the present study, Guffanti et al. found that the Kinect V2 was better at assessing angles in the sagittal plane compared to the coronal plane for gait analysis. They explained that this is attributed to the fact that the Kinect V2 relies on its depth sensor for assessing sagittal plane movement, such as knee flexion, while frontal plane movements have to rely on the spatial estimation of the camera alone, which might affect precision [10]. Galna et al. also reported that the Kinect has difficulty tracking more precise and fine movements such as toe tapping compared to gross movements such as sit-to-stand [16]. They explained that this may be due to the fact that the Kinect does not rely on manually placed surface markers, which may ultimately affect precision. Another potential explanation that has been cited is the rate at which the Kinect V2 captures data as opposed to the Vicon system [8]. The Kinect V2 device captures at a maximum rate of 30 Hz, or 30 frames per second [16]. The Vicon system, however, records at a rate of 120 Hz, which may result in increased precision when assessing the smaller coronal plane angles during explosive movement such as the DVJ [14]. Ultimately, the greater frame-to-frame variability of the Kinect V2 in the coronal plane has no clear clinical significance, as our results demonstrate that it accurately detects the specific angles associated with an increased risk of non-contact ACL injury when compared to the Vicon system.

### 4.4. Kinect V2 as a Screening Tool in Sports

Although originally designed for gaming applications, the Kinect V2 is equipped with infrared depth sensors and skeletal tracking that, when paired with the open-source Kinect V2 development kit, has proven to be convenient and inexpensive for kinematic imaging [17]. With the Kinect already being employed in healthcare applications, such as targeted rehabilitation programs for stroke patients and those suffering from Parkinson’s disease, the development of a screening tool for accurate biomechanical assessment is a logical extension of this concept [8,18,19,20]. As mobile health continues to grow and emerge as a field, sports teams would greatly benefit from incorporating this technology into the pre-season assessment of their athletes without requiring extensive resources. The portability of such a device makes it a practical tool for sports staff at all levels, and potentially provides technology comparable to high tech motion analysis systems that are traditionally only found in expensive research labs. Being able to quantify biomechanical deficits associated with ACL injuries could aid in identifying athletes at an increased risk of injury. Furthermore, establishing baseline data for healthy knees using this portable system would allow future comparisons and early detection for sports team staff. An additional future direction could include using the system to monitor progress during rehabilitation following ACL reconstruction surgery. Ultimately, the goal would be to get this technology into the hands of trainers and coaches who could identify and screen at-risk athletes who may benefit from sport-specific neuromuscular training to effectively decrease their risk of ACL injury.

### 4.5. Alternative Applications

The demonstrated ability of the Kinect V2 to track movement could potentially make it suitable for creating targeted rehabilitation programs for people recovering from conditions such as strokes or Parkinson’s disease, to name a few. Trained professionals could make use of such a system to monitor progress and provide real-time feedback. Additionally, this device has the potential to identify individuals who are at an increased risk of falling through the analysis of specific gait patterns and parameters. Lastly, this technology could be used as an aid in assessing different postural deviations of the spine, for example including scoliosis, by tracking body landmarks. This could have the potential to help in early intervention and treatment decisions.

### 4.6. Limitations

One of the potential limitations of this study was the relatively small sample size of six females and five males. After performing a total of three jumps each and examining each leg independently, this resulted in a total of 66 data points being assessed for each knee angle. Given the small sample size, differences between female and male participants were not analyzed. Future studies should be aimed at assessing a larger cohort of individuals to better quantify the accuracy and precision of the Kinect V2. It is also important to acknowledge that this study assessed healthy individuals and may not directly translate to individuals with a previous ACL injury. Future research is warranted to investigate the accuracy of the Kinect V2 in these populations to make the tool more generalizable.

Another potential limitation of this study is the inherent error associated with the Vicon system itself. Although the Vicon system is widely considered the gold standard motion analysis system, a study conducted by Merriaux et al. found that the Vicon system has a small, yet existent, degree of error [14]. In addition, the soft tissue movement between the skin markers used for the Vicon system and the underlying bone gives rise to a small degree of error as well [21]. Overall, we believe that these minor sources of inaccuracy are of limited clinical significance, as the Vicon system is commonly referred to as one of the gold standard motion analysis systems in the literature and has proven reliable in a wide variety of practical applications.

## 5. Conclusions

The Kinect V2 can measure specific DVJ parameters that are known to be associated with ACL injury risk with reliable accuracy. Specifically, ICC analysis demonstrates that the Kinect V2 measures IC, PC, and PS angles during a DVJ with excellent agreement compared to the current gold standard Vicon motion analysis system, with the added advantages of portability, ease of use, and a substantially lower cost. Further development of this tool, with a focus on the tracking of sagittal knee angles, may allow for the more widespread screening of athletes for ACL injury risk. This screening modality could ultimately benefit individuals at risk by enrolling them in validated ACL injury-prevention programs.

## Figures and Tables

**Table 1:** Participant characteristics ^α^.

Characteristic	Male Participants	Female Participants	All Participants
N	5	6	11
Age	20.7 ± 1.7	22.3 ± 2.9	21.6 ± 2.4
Height, m	1.8 ± 0.1	1.6 ± 0.1	1.7 ± 0.1
Weight, kg	75.0 ± 4.6	65.5 ± 12.4	69.8 ± 10.5
Body mass index	23.5 ± 3.7	23.2 ± 4.7	23.3 ± 4.1

^α^ Data are shown as mean ± SD.

**Table 2:** Comparison of mean DVJ parameters.

Knee Angle	Kinect V2 ^α^	Vicon System ^α^	Difference	*p* Value ^ß^
Left IC	−0.55 ± 5.66	−0.26 ± 5.64	−0.29	0.834
Right IC	−0.67 ± 5.15	−0.05 ± 5.47	−0.62	0.638
Left PC	6.74 ± 5.50	6.89 ± 5.34	−0.15	0.936
Right PC	5.32 ± 3.29	5.67 ± 3.60	−0.35	0.676
Left PS	115.14 ± 22.95	106.79 ± 22.48	8.35	0.140
Right PS	113.89 ± 21.61	106.15 ± 23.68	7.74	0.008

^α^ Data are shown as degrees ± SD. ^ß^ The Wilcoxon signed-rank test was used for the left PC and right PS. An independent-samples *t*-test was used for all other parameters.

**Table 3:** ICC analysis of DVJ parameters ^α^.

Knee Angle	ICC	95% Confidence Interval		*p* Value
		Lower Bound	Upper Bound	
Left IC	0.917	0.840	0.958	<0.001
Right IC	0.914	0.832	0.957	<0.001
Left PC	0.908	0.822	0.953	<0.001
Right PC	0.886	0.783	0.942	<0.001
Left PS	0.771	0.480	0.894	<0.001
Right PS	0.780	0.517	0.896	<0.001

^α^ Two-way mixed-effects model, single measures, absolute agreement.

## Data Availability

Data are available upon request from the authors.

## References

[1] Hewett T.E., Ford K.R., Xu Y.Y., Khoury J., Myer G.D. (2017). Effectiveness of neuromuscular training based on the neuromuscular risk profile. Am. J. Sports Med..

[2] Silvers-Granelli H.J., Bizzini M., Arundale A., Mandelbaum B.R., Snyder-Mackler L. (2017). Does the FIFA 11+ injury prevention program reduce the incidence of ACL injury in male soccer players?. Clin. Orthop. Relat. Res. ^®^.

[3] Yu B., Garrett W.E. (2007). Mechanisms of non-contact ACL injuries. Br. J. Sports Med..

[4] Boden B., Dean G., Feagin J., Garrett W. (1996). Mechanisms of ACL injury. Orthopedics.

[5] Hewett T.E., Myer G.D., Ford K.R., Heidt R.S., Colosimo A.J., McLean S.G., van den Bogert A.J., Paterno M.V., Succop P. (2005). Biomechanical measures of neuromuscular control and valgus loading of the knee predict anterior cruciate ligament injury risk in female athletes: A prospective study. Am. J. Sports Med..

[6] Hewett T.E., Roewer B., Ford K., Myer G. (2015). Multicenter trial of motion analysis for injury risk prediction: Lessons learned from prospective longitudinal large cohort combined biomechanical—Epidemiological studies. Braz. J. Phys. Ther..

[7] Corban J., Karatzas N., Zhao K.Y., Babouras A., Bergeron S., Fevens T., Rivaz H., Martineau P.A. (2023). Using an Affordable Motion Capture System to Evaluate the Prognostic Value of Drop Vertical Jump Parameters for Noncontact ACL Injury. Am. J. Sports Med..

[8] Gray A.D., Willis B.W., Skubic M., Huo Z., Razu S., Sherman S.L., Guess T.M., Jahandar A., Gulbrandsen T.R., Miller S. (2017). Development and Validation of a Portable and Inexpensive Tool to Measure the Drop Vertical Jump Using the Microsoft Kinect V2. Sports Health.

[9] Mokka Mokka: Motion Kinematic & Kinetic Analyzer. http://biomechanical-toolkit.github.io/docs/Mokka/index.html.

[10] Guffanti D., Brunete A., Hernando M., Rueda J., Navarro Cabello E. (2020). The accuracy of the microsoft kinect V2 sensor for human gait analysis. A different approach for comparison with the ground truth. Sensors.

[11] Mentiplay B.F., Hasanki K., Perraton L.G., Pua Y.-H., Charlton P.C., Clark R.A. (2018). Three-dimensional assessment of squats and drop jumps using the Microsoft Xbox One Kinect: Reliability and validity. J. Sports Sci..

[12] Mobini A., Behzadipour S., Saadat Foumani M. (2014). Accuracy of Kinect’s skeleton tracking for upper body rehabilitation applications. Disabil. Rehabil. Assist. Technol..

[13] Xu X., McGorry R.W., Chou L.-S., Lin J.-h., Chang C.-c. (2015). Accuracy of the Microsoft Kinect™ for measuring gait parameters during treadmill walking. Gait Posture.

[14] Merriaux P., Dupuis Y., Boutteau R., Vasseur P., Savatier X. (2017). A study of vicon system positioning performance. Sensors.

[15] Mousavi Hondori H., Khademi M. (2014). A Review on Technical and Clinical Impact of Microsoft Kinect on Physical Therapy and Rehabilitation. J. Med. Eng..

[16] Galna B., Barry G., Jackson D., Mhiripiri D., Olivier P., Rochester L. (2014). Accuracy of the Microsoft Kinect sensor for measuring movement in people with Parkinson’s disease. Gait Posture.

[17] Livingston M.A., Sebastian J., Ai Z., Decker J.W. Performance measurements for the Microsoft Kinect skeleton. Proceedings of the 2012 IEEE Virtual Reality Workshops (VRW).

[18] Park D.-S., Lee D.-G., Lee K., Lee G. (2017). Effects of virtual reality training using Xbox Kinect on motor function in stroke survivors: A preliminary study. J. Stroke Cerebrovasc. Dis..

[19] Shih M.-C., Wang R.-Y., Cheng S.-J., Yang Y.-R. (2016). Effects of a balance-based exergaming intervention using the Kinect sensor on posture stability in individuals with Parkinson’s disease: A single-blinded randomized controlled trial. J. Neuroeng. Rehabil..

[20] Vieira A., Gabriel J., Melo C., Machado J. (2017). Kinect system in home-based cardiovascular rehabilitation. Proc. Inst. Mech. Eng. Part H J. Eng. Med..

[21] Fiorentino N.M., Atkins P.R., Kutschke M.J., Goebel J.M., Foreman K.B., Anderson A.E. (2017). Soft tissue artifact causes significant errors in the calculation of joint angles and range of motion at the hip. Gait Posture.

